# Psychiatric symptoms and risk of victimisation: a population-based study from Southeast London

**DOI:** 10.1017/S2045796018000537

**Published:** 2018-09-10

**Authors:** V. Bhavsar, K. Dean, S. L. Hatch, J. H. MacCabe, M. Hotopf

**Affiliations:** 1 Department of Psychosis Studies, King's College London, Institute of Psychiatry, Psychology and Neuroscience, London, SE5 8AF, UK; 2 South London and Maudsley NHS Foundation Trust, Maudsley Hospital, London SE5 8AZ, UK; 3 School of Psychiatry, University of New South Wales, Australia; 4 Justice Health & Forensic Mental Health Network, New South Wales, Australia; 5 Department of Psychological Medicine, King's College London, Institute of Psychiatry, Psychology and Neuroscience, London, SE5 8AF, UK

**Keywords:** Common mental disorders, epidemiology, population survey, prospective study, violence

## Abstract

**Aims:**

Although violence is a vital public health problem, no prospective studies have tested for subsequent vulnerability to violence, as a victim or witness, in members of the general population with a range of psychiatric symptoms, or evaluated the importance of higher symptom burden on this vulnerability.

**Methods:**

We used successive waves of a household survey of Southeast London, taken 2 years apart, to test if association exists between psychiatric symptoms (symptoms of psychosis, common mental disorders, post-traumatic stress disorder and personality disorder) and later victimisation, in the form of either witnessing violence or being physically victimised, in weighted logistic regression models. Statistical adjustment was made for prior violence exposure, sociodemographic confounders, substance/alcohol use and violence perpetration. Sensitivity analyses were stratified by violence perpetration, sex and history of mental health service use.

**Results:**

After adjustments, psychiatric symptoms were prospectively associated with reporting any subsequent victimisation (odds ratio (OR) 1.88, 95% confidence interval (CI) 1.25–2.83), a two times greater odds of reporting witnessed violence (OR 2.24, 95% CI 1.33–3.76) and reporting physical victimisation (OR 1.76, 95% CI 1.01–3.06). One more symptom endorsed was accompanied by 47% greater odds of subsequent victimisation (OR 1.47, 95% CI 1.16–1.86). In stratified analyses, statistical associations remained evident in non-perpetrators, and among those without a history of using mental health services, and were similar in magnitude in both men and women.

**Conclusions:**

Psychiatric symptoms increase liability to victimisation compared with those without psychiatric symptoms, independently of a prior history of violence exposure and irrespective of whether they themselves are perpetrators of violence. Clinicians should be mindful of the impact of psychiatric symptoms on vulnerability to victimisation, including among those with common psychiatric symptoms and among those who are not considered at risk of perpetrating violence.

## Introduction

Violence towards people with psychiatric disorders remains a pressing public health and human rights issue (Krug *et al*., 2002), and negatively impacts symptoms in those with psychotic (Goodman *et al*., 1997) and bipolar disorders (Neria *et al*., 2005). Victimisation is also associated with greater service utilisation, greater substance misuse and poorer functional status in the community (Hodgins *et al*., 2009). The correlation between treated psychiatric disorders and suffering violence (as a witness and/or victim), particularly for physically violent victimisation, is supported by much observational evidence from large samples (Lehman and Linn, 1984; Hiday *et al*., 1999; Walsh *et al*., 2003; McDonald and Richmond, 2008; Maniglio, 2009), particularly among those with severe disorders such as schizophrenia. Various candidate explanations might contribute to this association. Victimisation could result in psychiatric disorder (Resnick *et al*., 1997; Acierno *et al*., 2002). Alternatively, the association could be influenced by risk factors for both victimisation and psychiatric disorder, for example, socioeconomic position (Wohlfarth *et al*., 2001) or substance misuse (Dansky *et al*., 1995; Hedtke *et al*., 2008). Thirdly, the association between psychiatric disorders and violence might be an artefact of selection bias, resulting from studying only people using services or who are being treated (Pearce and Richiardi, 2014). A further, less researched, possibility is that mental disorders themselves increase vulnerability to victimisation (Silver *et al*., 2005; Hart *et al*., 2012). In terms of these candidate explanations, there is now reasonable evidence for an effect of victimisation on mental disorder, particularly for disorders such as psychosis (in relation to childhood victimisation (Varese *et al*., 2012)) and post-traumatic stress disorder (PTSD) (Kessler *et al*., 1995; Kessler *et al*., 1999; Frissa *et al*., 2013). Recently, studies in general population cross-sectional samples have confirmed associations between victimisation and mental health (Kadra *et al*., 2014; Khalifeh *et al*., 2015), suggesting the association is not fully accounted for by selection biases affecting studies on clinical populations.

Prospective evidence on the relationship between mental disorders and later victimisation is needed. Whether increased vulnerability to victimisation applies only to more severe disorders such as psychosis, or also pertains to commoner psychiatric symptoms, is also unknown. Furthermore, violence perpetration and victimisation are known to overlap (Johnson *et al*., 2015, 2016); however, few studies of mental health and victimisation have accounted for violence perpetration (Silver *et al*., 2005; Choe *et al*., 2008).

Residents of Southeast London have high levels of psychiatric morbidity and mental health service use (Hatch *et al*., 2010, 2011). We have previously reported cross-sectional associations between victimisation and psychiatric symptoms (in particular, symptoms of depression/anxiety, psychotic symptoms, symptoms of post-traumatic stress (PTS) and personality symptoms). We found considerable overlap between the different forms of victimisation, and presented evidence that the distinction between proximal (i.e. in the last year) and distal (lifetime) violence exposure types revealed different patterns of sociodemographic and mental health associations (Kadra *et al*., 2014). In this previous study, the 1-year prevalence of witnessed violence was 7.4%, and of violent victimisation, 6.3%. This is substantially higher than the 1-year prevalence of physical violence reported by respondents to the 2007 British Crime Survey, a UK general population-based study of violence and crime, in which the proportion of respondents (aged 16 and over) reporting physical victimisation in the previous year was 2.4% (Kershaw *et al*., 2008). The relatively high levels of reported violence, and of psychiatric symptoms, in Southeast London, make it an appropriate setting to study the longitudinal association between psychiatric symptoms and violence.

Therefore, in this paper, we investigate the association between psychiatric symptoms and later victimisation (either by being physically victimised, or witnessing violence, and overall) in a representative sample of household residents in Southeast London. We hypothesise that the presence of psychiatric symptoms, and increasing number of symptom domains present, will be associated with later victimisation.

## Methods

### Sample details

The South East London Community Health study (SELCoH-1, 2008–2010) (Hatch *et al*., 2011) is a UK psychiatric and physical morbidity survey of 1698 adults aged 16 years and over, residing in 1075 randomly selected households in the boroughs of Southwark and Lambeth. Following SELCoH-1 (2008–2010), 1596 (94%) agreed to be re-contacted for a follow-up interview, of which 544 later declined consent for data collection or were ineligible due to death/poor health/relocation, The remaining 1052 participants (62%) were interviewed during 2011–2013, for SELCoH-2 (Hatch *et al*., 2016). Sampling was clustered by household, with all adults living in selected households invited to participate. Full details of the study, its sampling methods, and representativeness are published (Hatch *et al*., 2011). Data for this analysis on psychiatric symptoms were taken from SELCoH-1, and information on victimisation was drawn from SELCoH-2, and weights used to account for within-household non-response, clustering of responses within households and attrition between SELCoH-1 and SELCoH-2. Data on covariates for multivariable modelling were all taken from SELCoH-1.

### Measures

#### Psychiatric symptoms and service use at SELCoH-1

Psychiatric symptoms were measured in SELCoH-1 using a combination of community screening tools for separate domains of symptoms. The Psychosis Screening Questionnaire (PSQ) (Bebbington and Nayani, 1995) was used to assess non-affective psychotic symptoms, including strange experiences, paranoia, hallucinations and thought disorder. Individuals were considered to have psychotic symptoms if they endorsed one or more secondary items in these four areas. This approach is consistent with a previous analysis of psychotic symptoms originating from these data (Morgan *et al*., 2014). PTS symptoms were assessed using the PC-PTSD, a screening tool for PTSD designed for primary care use, which is based on the diagnostic criteria for PTSD in DSM-V. The PC-PTSD contains four items, of which three were necessary for the ascertainment of probable PTS symptoms – this cut-off identifies PTSD with a sensitivity of 0.76 and a specificity of 0.88 (Prins *et al*., 2003). The Clinical Interview Schedule (Revised, CIS-R (Lewis and Pelosi, 1990)), was used to measure symptoms of depression and anxiety, applying a cut-off score of 12 to identify those with depressive/anxiety symptoms, in line with the original receiver operating curve analysis and subsequent studies using this tool. The Standardised Assessment of Personality – Abbreviated Scale (SAPAS (Moran *et al*., 2003)) was used to identify people with probably personality dysfunction. This tool contains eight binary items assessing domains of personality function, of which four positive items were necessary to be coded as screening positive for personality dysfunction. The scale demonstrates good psychometric properties, and using a cut-off of 3, identifies the presence of personality disorder in clinical populations with a sensitivity of 0.94 and a specificity of 0.85. In accordance with previous research on non-clinical populations, a cut-off of 4 was used, which has a better positive predictive value in populations where the prevalence of clinically significant personality symptoms is lower (Fok *et al*., 2014).

Participants endorsing at least one of the domains: psychotic symptoms, depressive/anxiety symptoms, PTS symptoms or personality dysfunction, as defined above, were classed as having psychiatric symptoms in one or more domains. Participants were also classified based on the number of psychiatric symptom domains they endorsed, grouped into 0, 1–2 and 3–4 domains. Finally, a binary item was created for mental health service use, based on items assessing whether or not the respondent had seen a GP, mental health specialist or a psychological therapist for mental health reasons in the previous year.

#### SELCoH-1 covariates

Age was categorised in the following intervals: 16–24, 25–34, 35–54 and 55 years or older. Ethnicity information was available based on self-reported UK Census categories, which were collapsed into two categories reflecting white participants and those of black and minority ethnicity (BME). Employment status was categorised into employed, student, unemployed and other (Kadra *et al*., 2014). Recent use of illicit drugs was indicated by reports of the use of amphetamines, cocaine, crack cocaine, heroin, LSD or ecstasy in the previous year. A cut-off score of 8 out of 40 on the Alcohol Use Disorders Identification Test (Saunders *et al*., 1993) was used to identify hazardous alcohol consumption. Perpetration of violence was assessed by asking respondents whether they had ever (1) attacked or robbed someone; (2) injured someone with a weapon; or (3) hit, bit or slapped another person. This information was not available in SELCoH-2. Lifetime victimisation was assessed in SELCoH-1 by items inquiring whether the respondent had ever experienced (a) physical attack, (b) injury with a weapon, (c) witnessed violence or (d) either physical or sexual abuse in childhood. These were combined into a binary category reflecting any violence exposure at baseline.

#### Recent victimisation and witnessed violence

Respondents to SELCoH-2 were asked whether they had, in the previous 12 months, been exposed to physical violence in the form of having been attacked, robbed, mugged or been the victim of a serious crime; having been injured with a weapon, such as a gun, knife or stick; or been hit, bitten, slapped, kicked or sexually assaulted. Witnessed violence was determined by asking participants whether they had seen something violent happen to someone (e.g. someone being attacked or beaten or killed) in the last 12 months. Finally, a binary ‘overall victimisation’ variable was created, reflecting endorsement of either being physically victimised or witnessing violence.

### Analysis

All statistical analyses were performed in STATA 14 (StataCorp, 2014), and took account of weights for non-response within households, household clustering and attrition between SELCoH-1 and SELCoH-2. Participants successfully interviewed at SELCoH-2 were compared with those not interviewed (for reasons of ineligibility or loss to follow-up). Univariate associations were estimated between the endorsement of each psychiatric symptom domain and each violence type, using logistic regression models, estimating odds ratios. For multivariable logistic regression modelling, potential confounders selected from the literature were included as covariates if the difference between the adjusted and unadjusted estimates was >10% (Greenland *et al*., 2016). Having identified potential confounders for inclusion in the final model, these were grouped into baseline victimisation (model I), then adding sociodemographic confounders (model II) and then substance-related confounders (model III). Finally, we additionally adjusted for perpetration to arrive at a fully adjusted estimate (model IV).

Linear associations between the number of psychiatric symptom domains endorsed, and odds of victimisation, were assessed with likelihood ratio tests. To further assess the influence of gender, perpetration history and evaluate whether associations were limited to those with previous mental health service use, further analyses were stratified by perpetration history in S1, history of mental health service use and sex.

## Results

### Description of sampling

Of 1698 individuals participating in SELCoH-1, 1052 (62%) participated in SELCoH-2 (Hatch *et al*., 2016). Respondents in the first wave of data collection who were lost to follow-up tended to be younger, male, unemployed and of BME ethnicity. The time elapsed between baseline and follow-up data collection was 3 years. Interval between the baseline and follow-up interview ranged between 14.8 and 51.8 months, with a median of 29.9 months; this did not vary statistically by victimisation/witness status.

### Included participants

After excluding 54 records with missing data on any modelled variables in both waves of data collection, 998 participants remained, with an age range of 16–88, of whom 59% were female and 35% were of BME ethnicity. Three hundred and sixty-nine participants (37%, Table 1) endorsed any psychiatric symptom domain at baseline interview. Meeting thresholds for one or more of the symptom domains was commoner in women than men, and among the unemployed compared with the employed. Endorsing one or more symptom domain was around twice as common among those with a history of service use (62%), compared with those without (34%). In baseline data, PTS symptoms were least prevalent and depressive/anxiety symptoms most common. Nearly three-fifths had been exposed to violence during their lifetime in the baseline interview. At follow-up, 5.9% of participants reported recent (past 12 months) physical victimisation, 6.8% reported recent witnessed violence and 11.2% reported any victimisation (Table 2).

**Table 1.** Descriptive data on included participants classified by presence of any psychiatric symptom domain (*n* = 998)

	No psychiatric symptom domain	Any psychiatric symptom domain
Age		
16–24	108 (57.6)	79 (42.4)
25–34	140 (63.2)	79 (36.8)
35–44	139 (67.5)	65 (32.5)
45–54	107 (60.4)	68 (39.6)
55+	135 (62.6)	78 (37.4)
Gender		
Male	271 (64.8)	142 (35.2)
Female	358 (59.7)	227 (40.3)
Employment status		
Employed	404 (69.6)	175 (30.4)
Student	77 (57.0)	60 (43.0)
Unemployed	39 (44.3)	47 (55.7)
Other	109 (53.6)	87 (46.4)
Ethnicity		
White	409 (61.8)	244 (38.2)
BME	220 (62.9)	124 (37.1)
Mental health service use		
No	573 (66.2)	279 (33.8)
Yes	56 (37.8)	90 (62.2)
Hazardous alcohol use		
No	515 (63.7)	281 (36.3)
Yes	114 (56.7)	88 (43.3)
Recent drug use		
No	521 (65.1)	272 (34.9)
Yes	108 (52.4)	97 (47.6)
Lifetime exposure to any violence at baseline		
No	291 (75.9)	90 (24.0)
Yes	338 (54.2)	279 (45.8)
Lifetime violence perpetration		
No	459 (69.1)	200 (30.9)
Yes	170 (50.1)	169 (49.9)
Total	629 (62.1)	369 (37.9)

BME, black and minority ethnic status.

Raw counts are presented, with survey-weighted proportions in parentheses.

**Table 2.** Descriptive data on overall sample, and included participants

	Overall (*n* = 1698, 100%)	Included in analysis^a^ (*n* = 998, 59%)	Recently physically victimised at follow-up^b^ (*n* = 58, 5.9%)	Recently witnessed violence at follow-up^b^ (*n* = 72, 6.8%)	Any victimisation at follow-up^b^ (*n* = 116, 11.2%)
**Age**					
** 16–24**	**356**	187 (52.5)	23 (12.9)	24 (14.0)	41 (23.5)
** 25–34**	**404**	219 (54.2)	14 (6.4)	17 (8.4)	27 (12.6)
** 35–44**	**336**	204 (60.7)	9 (4.5)	10 (5.2)	17 (8.6)
** 45–54**	**164**	175 (66.3)	4 (2.3)	10 (6.1)	14 (8.4)
** 55+**	**338**	213 (63.0)^b^	8 (3.6)	11 (5.2)	17 (7.9)
**Gender**					
** Male**	**737**	413 (56.0)	25 (7.1)	45 (12.0)	62 (16.7)
** Female**	**961**	585 (60.9)^c^	33 (6.1)	27 (5.0)	54 (9.9)
**Employment status**					
** Employed**	**921**	579 (62.9)	32 (6.0)	34 (6.3)	60 (11.1)
** Student**	**247**	137 (55.5)	13 (10.3)	22 (17.5)	31 (24.6)
** Unemployed**	**170**	86 (50.6)	7 (8.9)	8 (9.4)	11 (13.5)
** Other**	**351**	196 (55.8)^d^	6 (3.1)	8 (4.7)	14 (7.8)
**Ethnicity**					
** White**	**1051**	654 (62.1)	38 (6.6)	39 (6.5)	67 (9.7)
** BME**^**a**^	**645**	344 (53.3)^e^	21 (6.6)	33 (11.2)	49 (15.9)
**Any psychiatric symptom domain**					
** No**	**1050**	629 (59.9)	26 (4.7)	31 (5.8)	54 (10.0)
** Yes**	**648**	369 (56.9)^f^	32 (9.6)	41 (12.4)	62 (18.3)
**Personality dysfunction**					
** No**	**1421**	859 (60.5)	43 (5.9)	56 (7.6)	90 (12.1)
** Yes**	**241**	139 (57.7)^g^	15 (10.5)	16 (12.5)	26 (19.3)
**Psychotic symptoms**					
** No**	**1367**	821 (60.1)	42 (5.8)	48 (6.9)	81 (11.9)
** Yes**	**323**	177 (54.8)^h^	16 (9.8)	24 (14.1)	35 (21.0)
**Post-traumatic stress symptoms**					
** No**	**1591**	948 (59.6)	55 (6.6)	63 (7.8)	105 (12.6)
** Yes**	**89**	50 (56.2)^i^	3 (6.5)	9 (18.5)	11 (22.8)
**Depressive/anxiety symptoms**					
** No**	**1296**	766 (59.1)	41 (6.0)	41 (7.7)	83 (12.4)
** Yes**	**396**	232 (58.6)^j^	17 (8.3)	22 (10.5)	33 (15.5)
**Mental health service use**					
** No**	**1507**	852 (58.1)	48 (6.1)	60 (7.7)	98 (12.3)
** Yes**	**191**	122 (63.9)^k^	10 (9.1)	12 (11.8)	18 (17.9)
**Hazardous alcohol use**					
** No**	**1344**	796 (59.2)	56 (5.7)	40 (8.4)	88 (12.8)
** Yes**	**343**	202 (58.9)^l^	16 (9.5)	18 (8.1)	28 (14.5)
**Recent drug use**					
** No**	**1329**	793 (59.7)	35 (4.9)	48 (6.9)	88 (11.3)
** Yes**	**363**	205 (56.5)^m^	23 (11.9)	24 (12.8)	28 (19.2)
**Lifetime exposure to any violence at baseline**					
** No**	**673**	381 (56.6)	18 (4.9)	13 (3.8)	30 (9.5)
** Yes**	**1025**	617 (60.2)^n^	40 (7.5)	59 (10.9)	86 (15.8)
**Lifetime violence perpetration**					
** No**	**1139**	659 (57.9)	26 (4.1)	36 (6.5)	57 (9.6)
** Yes**	**540**	339 (62.8)^o^	32 (10.7)	36 (11.5)	59 (19.2)

BME, black and minority ethnic status.

Raw counts are presented, with survey-weighted proportions in parentheses.

a Percentages based on proportion of baseline sample (*n* = 1698).

b Percentages based on proportion of participants included in the analysis (*n* = 998).

χ^2^ *p*-value for the association with inclusion in analysis: ^b^*p* = 0.001, ^c^*p* = 0.045, ^d^*p* = 0.004, ^e^*p* < 0.001, ^f^*p* = 0.229, ^g^*p* = 0.416, ^h^*p* = 0.084, ^i^*p* = 0.524, ^j^*p* = 0.854, ^k^*p* = 0.129, ^l^*p* = 0.911, ^m^*p* = 0.273, ^n^*p* = 0.142, ^o^*p* = 0.055.

### Univariate associations

Victimisation reduced with age, and was more common among males, those of BME ethnicity, those reporting recent substance use and those meeting thresholds for any psychiatric symptom domains (Table 3). Overall victimisation was statistically associated with psychotic symptoms, but not with personality dysfunction, PTS symptoms or depressive/anxiety symptoms, after adjusting for prior violence exposure. Psychotic symptoms and hazardous alcohol use were associated with subsequent witnessed violence, after adjustment for prior violence exposure.

**Table 3.** Univariate prospective associations (odds ratios with 95% confidence intervals) with each type of violence exposure in the final sample (*n* = 998)

	Recent physical victimisation	Recent physical victimisation^a^	Recent witnessed violence	Recent witnessed violence^a^	Any recent victimisation	Any recent victimisation^a^
Age in years^b^						
25–34	0.56 (0.29–1.10)	0.56 (0.28–1.12)	0.46 (0.22–0.97)	0.46 (0.22–0.97)	0.47 (0.27–0.83)	0.47 (0.26–0.83)
35–44	0.33 (0.15–0.73)	0.34 (0.15–0.75)	0.32 (0.15–0.71)	0.33 (0.15–0.72)	0.31 (0.17–0.57)	0.31 (0.17–0.58)
45–54	0.40 (0.18–0.88)	0.38 (0.17–0.85)	0.16 (0.05–0.47)	0.15 (0.05–0.47)	0.30 (0.15–0.60)	0.29 (0.14–0.58)
55+	0.33 (0.15–0.73)	0.34 (0.15–0.74)	0.25 (0.11–0.57)	0.25 (0.11–0.57)	0.28 (0.15–0.52)	0.28 (0.15–0.52)
Male	1.19 (0.68–2.07)	1.09 (0.63–1.90)	2.58 (1.51–4.41)	2.20 (1.27–3.83)	1.82 (1.20–2.76)	1.63 (1.06–2.50)
Unemployed	1.46 (0.66–3.22)	1.38 (0.63–3.00)	1.16 (0.51–2.63)	1.02 (0.45–2.33)	1.03 (0.54–1.98)	0.95 (0.50–1.79)
BME ethnicity	1.80 (1.05–3.08)	1.86 (1.08–3.19)	1.00 (0.56–1.80)	1.01 (0.56–1.82)	1.47 (0.952.28)	1.49 (0.96–2.33)
Any psychiatric symptom domain	2.17 (1.24–3.80)	2.04 (1.14–3.66)	2.27 (1.38–3.74)	1.92 (1.16–3.17)	2.02 (1.36–3.00)	1.81 (1.2–2.71)
Psychotic symptoms	1.76 (0.94–3.31)	1.62 (0.85–3.07)	2.20 (1.31–3.70)	1.81 (1.07–3.05)	2.09 (1.34–3.26)	1.84 (1.18–2.87)
Personality dysfunction	1.88 (0.98–3.59)	1.76 (0.91–3.41)	1.74 (0.96–3.17)	1.51 (0.83–2.75)	1.73 (1.04–2.86)	1.57 (0.94–2.60)
Post-traumatic stress symptoms	0.98 (0.31–3.12)	0.87 (0.28–2.73)	2.70 (1.23–5.95)	2.14 (0.97–4.73)	2.04 (1.01–4.14)	1.73 (0.85–3.51)
Depressive/anxiety symptoms	1.41 (0.75–2.65)	1.31 (0.69–2.51)	1.41 (0.80–2.48)	1.31 (0.69–2.51)	1.29 (0.80–2.07)	1.15 (0.70–1.89)
Mental health service use	1.53 (0.78–3.02)	1.47 (0.74–2.92)	1.59 (0.86–2.96)	1.47 (0.79–2.76)	1.55 (0.91–2.64)	1.47 (0.86–2.51)
Hazardous alcohol use	1.74 (1.00–3.03)	1.65 (0.94–2.90)	0.96 (0.53–1.75)	3.15 (1.62–6.14)	1.16 (0.74–1.82)	1.06 (0.67–1.70)
Recent drug use	2.64 (1.51–4.61)	2.50 (1.43–4.37)	1.99 (1.12–3.54)	1.70 (0.96–3.03)	1.88 (1.15–3.07)	1.69 (1.03–2.77)
Lifetime exposure to violence at baseline	1.59 (0.88–2.86)	–	3.10 (1.61–5.97)	−	2.02 (1.29–3.18)	−
Lifetime violence perpetration	2.76 (1.59–4.80)	2.69 (1.50–4.80)	1.88 (1.15–3.09)	1.41 (0.83–2.42)	2.22 (1.46–3.39)	1.92 (1.21–3.03)

All estimates are based on 998 cases with complete records on modelled variables, take account of household non-response, household clustering of responses and attrition between baseline and follow-up interviews.

a Adjusted for lifetime violence exposure at baseline.

b Reference group is 16–24, the youngest age group.

### Multivariable modelling

After adjustment, endorsing any psychiatric symptom domain was associated with a greater than twofold increase in the odds of later witnessed violence, a 1.75-fold increase in the odds of being physically victimised and a close to twofold increase in the odds of overall victimisation (see Table 4). Statistical evidence (*p* < 0.001) was found for a linear trend between the number of symptom domains endorsed and overall victimisation. Compared with those not endorsing any symptom domain, those reporting 3–4 symptom domains had more than three times the odds of reporting recently witnessed violence, and twice the odds of reporting physical victimisation, in adjusted models. For each further symptom domain endorsed, there was a 1.55-fold increase in the odds of later witnessed violence, a 1.3-fold increase in the odds of later physical victimisation, and a 1.47-fold increase in the odds of overall victimisation.

**Table 4.** Partial and fully adjusted logistic regression models for the association (odds ratios with 95% confidence intervals) between psychiatric symptom domains in S1 interview and recent exposure to violence at follow-up

	Baseline psychiatric symptom domains	Model I^a^	Model II^b^	Model III^c^	Model IV^d^
Recently witnessed violence at follow-up	Any psychiatric symptom domain^e^	1.92 (1.16–3.17)	2.09 (1.26–3.48)	2.30 (1.39–3.83)	2.24 (1.33–3.76)
	1–2 domains^e^	1.80 (1.06–3.05)	1.96 (1.15–3.34)	2.12 (1.25–3.61)	2.08 (1.21–3.56)
	3–4 domains^e^	2.49 (1.12–5.53)	2.92 (1.21–7.05)	3.52 (1.46–8.48)	3.35 (1.36–8.29)
	One more domain	1.39 (1.09–1.77)	1.48 (1.14–1.93)	1.58 (1.22–2.05)	1.55 (1.19–2.03)^f^
Recently physically victimised at follow-up	Any psychiatric symptom domain^e^	2.04 (1.14–3.66)	1.98 (1.10–3.57)	1.94 (1.11–3.40)	1.76 (1.01–3.06)
	1–2 domains^e^	1.95 (1.05–3.61)	1.89 (1.01–3.52)	1.85 (1.02–3.37)	1.72 (0.95–3.10)
	3–4 domains^e^	2.53 (0.91–7.05)	2.57 (0.90–7.36)	2.48 (0.90–6.80)	2.00 (0.71–5.62)
	One more domain	1.41 (1.06–1.88)	1.42 (1.05–1.92)	1.40 (1.06–1.86)	1.31 (0.97–1.75)^f^
Any victimisation at follow-up	Any psychiatric symptom domain^e^	1.81 (1.21–2.72)	1.90 (1.26–2.88)	2.01 (1.35–3.01)	1.88 (1.25–2.83)
	1–2 domains^e^	1.66 (1.09–2.52)	1.74 (1.14–2.67)	1.82 (1.20–2.76)	1.72 (1.13–2.63)
	3–4 domains^e^	2.65 (1.31–5.38)	3.03 (1.40–6.55)	3.36 (1.58–7.13)	2.94 (1.35–6.41)
	One more domain	1.41 (1.13–1.76)	1.48 (1.17–1.88)	1.54 (1.22–1.93)	1.47 (1.16–1.86)^f^

All estimates take account of clustering of responses within households and household non-response, and are based on 998 cases with complete data on all modelled variables. For each outcome, we present three specifications of psychiatric symptom domain – a binary outcome (any psychiatric symptom domain endorsed, compared with no psychiatric symptom domain endorsed), categorisations into 0, 1–2 and 3–4 symptom domains endorsed, and a linear model based on number of symptom domains endorsed.

a Model I is adjusted only for lifetime violence exposure at baseline.

b Model II is further adjusted for age (continuous), gender, ethnicity and unemployment.

c Model III is further adjusted for hazardous alcohol use and recent drug use.

d Model IV is further adjusted for perpetration.

e Reference group for these comparisons is the group with no psychiatric symptom domains.

f Likelihood ratio tests for significance of linear trend in number of symptom domains was <0.001 in all fully adjusted models for witnessed violence, victimisation and any violence exposure.

In order to examine the influence of important potential confounders in more depth, we repeated the analyses shown in Table 3 stratifying by perpetration status, sex and history of service use (Table 4). Table 5 presents estimates for the association between any psychiatric symptom domain and any subsequent victimisation, stratified by perpetration history, gender and mental health service use. Endorsing any psychiatric symptom domain remained prospectively associated with overall victimisation both in those with and without a history of perpetration, and among women and men, however the association among women was greater in magnitude, and the confidence interval for the final estimate in men crossed null. The association between any psychiatric symptom domain and later victimisation was greater among those with a history of service use than those without, where it remained, but was statistically significant in both groups. Statistical evidence for a linear relationship between number of psychiatric symptom domains and odds of later victimisation was evident both in those with and without a history of service use, and among non-perpetrators, in men and in women. However, fully adjusted estimates for perpetrators no longer produced statistical evidence of an association.

**Table 5.** Estimates for the association between psychiatric symptom domains endorsed and any later violence exposure, limited to those with and without a lifetime history of perpetration, to those with and without a history of mental health service use, and to men and women

Models restricted to:		Model I	Model II	Model III	Model IV
Non-perpetrators (based on 659 participants)	Any psychiatric symptom domain^a^	1.51 (0.86–2.66)	1.64 (0.93–2.90)	1.77 (1.00–3.13)	–
	1-2 domains^a^	1.24 (0.67–2.29)	1.35 (0.73–2.51)	1.44 (0.77–2.69)	–
	3–4 domains^a^	4.37 (1.57–12.19)	4.82 (1.66–14.01)	5.38 (1.95–14.88)	–
	One more domain	1.55 (1.10–2.20)	1.62 (1.14–2.28)	1.68 (1.21–2.34)	–
Perpetrators (339)	Any psychiatric symptom domain^a^	1.88 (1.06–3.32)	2.01 (1.10–3.66)	1.90 (1.04–3.48)	–
	1–2 domains^a^	1.91 (1.08–3.37)	2.03 (1.12–3.67)	1.91 (1.05–3.47)	–
	3–4 domains^a^	1.78 (0.67–4.71)	1.90 (0.62–5.86)	1.87 (0.58–6.01)	–
	One more domain	1.25 (0.94–1.66)	1.29 (0.93–1.80)	1.28 (0.91–1.80)	–
Men (413)	Any psychiatric symptom domain^a^	1.69 (0.98–2.91)	1.69 (0.96–2.95)	1.75 (0.99–3.07)	1.65 (0.93–2.92)
	1–2 domains^a^	1,46 (0.82–2.60)	1.47 (0.81–3.78)	1.49 (0.83–2.69)	1.41 (0.77–2.57)
	3–4 domains^a^	3.50 (1.33–9.25)	3.78 (1.20–11.94)	4.13 (1.29–13.22)	3.88 (1.21–12.41)
	One more domain	1.51 (1.10–2.06)	1.54 (1.08–2.19)	1.58 (1.11–2.25)	1.54 (1.07–2.20)
Women (585)	Any psychiatric symptom domain^a^	2.38 (1.24–4.56)	2.30 (1.20–4.40)	2.49 (1.35–4.61)	2.27 (1.21–4.27)
	1–2 domains^a^	2.27 (1.16–4.44)	2.21 (1.14–4.28)	2.38 (1.25–4.52)	2.23 (1.17–4.26)
	3–4 domains^a^	2.92 (1.07–7.98)	2.81 (0.94–8.38)	3.11 (1.12–8.61)	2.53 (0.83–7.70)
	One more domain	1.46 (1.08–1.97)	1.45 (1.04–2.02)	1.52 (1.12–2.06)	1.42 (1.01–1.98)
Participants with no history of service use (852)	Any psychiatric symptom domain^a^	1.62 (1.06–2.49)	1.62 (1.03–2.57)	1.71 (1.09–2.68)	1.62 (1.03–2.56)
	1–2 domains^a^	1.58 (1.01–2.45)	1.59 (1.00–2.51)	1.66 (0.05–2.61)	1.59 (1.01–2.51)
	3–4 domains^a^	1.93 (0.76–4.89)	1.93 (0.62–5.98)	2.15 (0.73–6.38)	1.91 (0.60–6.01)
	One more domain	1.31 (1.01–1.70)	1.32 (0.97–1.81)	1.38 (1.02–1.85)	1.32 (0.97–1.81)
Participants with a history of service use (146)	Any psychiatric symptom domain^a^	2.83 (0.76–10.51)	2.69 (0.76–9.55)	2.79 (0.90–8.62)	2.47 (0.76–8.03)
	1–2 domains^a^	2.32 (0.57–9.45)	2.19 (0.57–8.49)	2.15 (0.63–7.32)	2.00 (0.57–7.00)
	3–4 domains^a^	4.95 (1.06–23.17)	5.30 (1.16–24.22)	5.29 (1.43–19.64)	4.46 (1.09–18.20)
	One more domain	1.62 (1.06–2.49)	1.69 (1.06–2.67)	1.70 (1.13–2.56)	1.60 (1.03–2.49)

All models are based on 998 participants with complete data for the modelled variables and weighted for household non-response at both waves. Models are numbered as in Table 3. ^a^Reference group is no symptom domain endorsed. ^b^*p* = 0.006, ^c^*p* = 0.105, ^d^*p* = 0.018, ^e^*p* = 0.035, ^f^*p* = 0.064, ^g^*p* = 0.028.

## Discussion

### Summary of findings

In a sample of Southeast London household residents, psychiatric symptoms, ascertained based on endorsement of epidemiological screening tools for different domains of psychopathology, were prospectively associated with later victimisation over a 3-year period, both overall, by being physically victimised, and as a witness, compared with those without symptoms at baseline. An increasing number of symptom domains predicted greater odds of victimisation over time. The association was not limited to perpetrators of violence, or to those without a history of mental health service use. Although associations between endorsing any psychiatric symptom domain and later victimisation were observed in both men and women, estimates for women were of greater magnitude.

### What this study adds

We suggest that our study significantly strengthens a limited literature (Silver *et al*., 2005; Hart *et al*., 2012) pointing to an association between a range of psychiatric symptoms and later victimisation, confirming this in a longitudinal population-based sample. In our study, recent (12-month) victimisation at follow-up was reported by 9.6% of people reporting any psychiatric symptom domain, and 4.7% in those without, comparing favourably with other estimates (Maniglio, 2009). The study also contributes by using data drawn from a representative sample of household residents who were not using mental health services, and includes information on witnessed violence as well as violent victimisation. Our evidence that psychiatric symptoms were associated with later witnessed violence, together with evidence of the psychiatric sequelae of witnessed violence (Fitzpatrick and Boldizar, 1993), implies a bi-directional relationship between witnessed violence and psychiatric symptoms that warrants further examination. In addition to adjusting for gender in regression models in line with previous work (Silver *et al*., 2005; Hart *et al*., 2012), we found evidence for a stronger association among women in stratified analyses, and evidence for association even in those not using mental healthcare, as well as perpetrators of violence.

### Previous literature

The psychiatric consequences of victimisation are well known, and include psychosis (Varese *et al*., 2012), depression (Dorrington *et al*., 2014) and PTSD (Liu *et al*., 2017). Previous evidence on increased victimisation in people with psychiatric disorders have been based on cross-sectional and case–control designs (Kamperman *et al*., 2014; Rodway *et al*., 2014; Tsigebrhan *et al*., 2014; Meijwaard *et al*., 2015) – we demonstrate this association in prospective data. Moreover, previous studies have been confined to clinical populations with severe mental disorder (Bebbington *et al*., 2000; Alonso *et al*., 2007; Howard *et al*., 2010), have not directly sampled the general population for controls (Brennan *et al*., 2010; Rodway *et al*., 2014), have been cross-sectional in design (Sturup *et al*., 2011; Desmarais *et al*., 2014; Kamperman *et al*., 2014; Tsigebrhan *et al*., 2014; Meijwaard *et al*., 2015), have not examined the association of psychiatric symptoms with witnessing violence and have not accounted for perpetration history (Stickley and Carlson, 2010; Desmarais *et al*., 2014; Meijwaard *et al*., 2015). Hart *et al.* found prospective association between a single scale reflecting psychiatric morbidity and violent experiences, but examined only individuals remaining in the study at age 46 (Hart *et al*., 2012), which is not the peak age for victimisation experiences. They did not distinguish between different psychiatric disorders in their data, lacked information on perpetration and did not account for victimisation occurring prior to the development of psychiatric disorder. Honings *et al*. (2017) reported evidence of bi-directional associations between psychiatric symptoms and victimisation based on prospective data from the Netherlands, however their analysis was limited to psychotic symptoms, and did not directly assess perpetration of violence (instead adjusting for history of overall arrest).

### Strengths and limitations

This study was longitudinal and based on a randomly selected baseline sample. Detailed measurements of psychopathology were gathered, and we used conservative cut-offs to identify individuals in whom we could be reasonably confident there were clinically relevant symptoms in the various domains. On the other hand, there was attrition, which reduced the precision of estimates and limited study power to estimate associations with specific symptom domains in detail, as planned. People with psychiatric symptoms might have been more or less liable to report victimisation compared with people without psychiatric symptoms, leading to misclassification and resulting over or underestimation of the main association. However, studies indicate that the recall of victimisation events is generally reliable (Schneider, 1981; Goodman *et al*., 1999), and our investigation of victimisation events focused on events in the previous year. Information on perpetration was only available at one time point. We did not have information on the number and intensity of violent experiences, which is a pressing need in public health research (Krieger, 2012; Walby *et al*., 2017). Because our two waves of data collection took place within 3 years of each other, we were unable to assess longer term consequences of psychiatric symptoms in these data, in contrast to some previous studies (Silver *et al*., 2005; Hart *et al*., 2012). Although we adjusted estimates for prior victimisation in order to limit confounding, it is also possible that we overadjusted our estimates in this study (Glymour *et al*., 2005). Finally, baseline survey respondents lost to follow-up tended to be younger, male, unemployed and more commonly of BME ethnicity compared with those whose were successfully followed up, leaving open the possibility of selection bias. Given that younger age, male gender and BME ethnicity were associated with victimisation in this study, consistent with other evidence (Brennan *et al*., 2010), it is likely that bias introduced into our estimates through biased attrition deviated our estimates towards, rather than away from the null. Although we were able to examine a wider range of psychiatric symptoms than previous studies, it was not possible to include all psychiatric symptoms; in principle, other symptom categories, not measured in this study, could display opposite associations with later victimisation. We would caution against generalizing these results to psychiatric symptoms not measured in this study. This analysis was based on two waves of a household survey, with some loss to follow-up attrition between the waves. We did not have information on the precise timing of offences, or time of loss to follow-up, and our analysis is therefore based only on individuals on whom data were collected in the second wave.

Although our results suggest that psychiatric symptoms may increase liability to subsequent victimisation, the exact explanations remain unclear. Our findings may, for example, be consistent with a ‘routine activities’ model of victimisation where violent experiences arise from the convergence of motive, opportunity and lack of adequate safeguards against violence (Miethe *et al*., 1987). Psychiatric disorders are socially and culturally stigmatising, which might lead to increasing conflict in daily life (Cohen and Felson, 1979; Link *et al*., 1999), however our study had no information on the perpetrators of violence experienced by survey respondents. Psychiatric symptoms not measured in this study, such as irritability, social withdrawal or disorganised behaviour, could increase risk of attack from other people (Brekke *et al*., 2001; Walsh *et al*., 2003; Fortugno *et al*., 2013; de Mooij *et al*., 2015). One study has suggested that victimisation risk in people with psychiatric disorder is related to the experience of financial stress (Honkonen *et al*., 2004), on which information was also unavailable. Hazardous use of substances and alcohol are other potential mediators (Schomerus *et al*., 2008; Dolan *et al*., 2012), which is consistent with the attenuation of estimates seen upon adjustment in the present study. Finally, there is strong evidence that repeated victimisation experiences tend to cluster in individuals over time (Goodman *et al*., 2001; Cotter *et al*., 2016; Pridemore and Berg, 2017); in a prospective study of people with psychosis, reporting assault was associated with prior victimisation, early illness onset, infrequent family contact and personality difficulties, implying that early life adversity might play a role in patterning social interactions over the life course, and result in the emergence of victimisation, enduring dysfunctional personality traits, and psychosis (Dean *et al*., 2007). This evidence implies the presence of underlying factors driving victimisation in particular individuals, for whom diagnosis and treatment may have a limited impact. We adjusted estimates for prior instances of violence exposure as a way of accounting not only for the direct effects of prior violence exposure on later violence (through aberrant coping, e.g.), but also for sociodemographic and other risk factors for the earlier exposure to violence. The suggestion from our results that the association between psychiatric symptoms and later victimisation is greater among women requires further investigation.

## Conclusions

We present the first prospective evidence that people with common psychiatric symptoms, and higher number of symptoms, have greater vulnerability to victimisation than those without symptoms, not limited to those with a history of perpetrating violence, those using services or those with prior exposure to violence. Lifestyle factors such as hazardous alcohol use and drug use, as well as perpetration history, appear to account for some of this association. There is already evidence that people with psychiatric disorders are systematically excluded from the benefits of public health interventions addressing, for example, smoking (Szatkowski and McNeill, 2014) and healthy eating (Cabassa *et al*., 2010). We tentatively suggest that this might also be true for violence prevention programmes, safer neighbourhood interventions and policing. Clinicians and health services have a role in maintaining the personal safety of people with mental illness (Manthorpe and Martineau, 2010). Clinicians should be mindful of the impact of psychiatric symptoms on vulnerability to victimisation, including among those with common psychiatric symptoms, such as depression, and among those who are not considered at risk of perpetrating violence.

## Availability of data and materials

The data that support the findings of this study are available from Professor Matthew Hotopf but restrictions apply to the availability of these data, which were used under license for the current study, and so are not publicly available. Data are however available from the authors upon reasonable request and with permission of Professor Matthew Hotopf.
